# Breaking boundaries: the transformative role of exercise in managing multiple sclerosis

**DOI:** 10.17179/excli2024-6932

**Published:** 2024-04-15

**Authors:** Saber Saedmocheshi, Narimen Yousfi, Karim Chamari

**Affiliations:** 1 Department of Physical Education and Sport Sciences, Faculty of Humanities and Social Sciences, University of Kurdistan, Sanandaj, Iran; 2 Tunisian Research Laboratory "Sport Performance Optimisation", (LR09SEP01) National Center of Medicine and Science in Sport, Tunis, Tunisia; 3 Higher Institute of Sport and Physical Education, ISSEP Ksar Said, Manouba University, Tunis, Tunisia

**Keywords:** multiple sclerosis, flexibility training, immunomodulation, rehabilitation, quality of life, physical activity

## Abstract

Multiple sclerosis (MS) is a prevalent cause of physical disability in adults, with inflammation-induced demyelination and neurodegeneration contributing to its etiology. This comprehensive review explores the multifaceted benefits of exercise in managing MS, including improvements in aerobic capacity, balance, muscle strength, immune and hormonal functions and mood. Various exercise modalities, such as aerobic, resistance, flexibility, and balance training, are discussed, along with tailored protocols for MS patients. Recommended exercise strategies are: aerobic exercise: 2-3x/week; 10-30 minutes (40 %-60 % of maximum heart rate (HR_max_), HIIT: 1x/week, five 30-90-second intervals at 90 %-100 % HR_max_, Resistance training: 2-3x/week, 5-10 exercises; 1-3 sets for each exercise, 8-15 repetitions/set. The review also examines the impact of exercise on neuroplasticity, cardiovascular responses, cytokine modulation, stress hormone regulation, brain structure, and function and fatigue perception. Emphasizing the importance of exercise in enhancing the quality of life for individuals with MS, the review proposes exercise prescriptions and highlights the promising link between physical activity, brain health, and improved hormonal and immune status in MS patients. This review aims to inform future research and guide clinical practices for effective MS management.

## Introduction

Multiple sclerosis (MS) stands as one of the most common diseases associated with physical disability in adults (Hauser and Cree, 2020[53]). Its increasing prevalence in contemporary societies remains an enigma, with the exact reasons behind this surge yet to be fully elucidated (McGinley et al., 2021[82]). Research suggests that a complex interplay of genetic and environmental factors contributes to the development of MS (Dobson and Giovannoni, 2019[26]). This multifaceted disease is characterized by two primary features 1) inflammation leading to demyelination of neurons, and 2) astroglial proliferation (gliosis) and neurodegeneration, primarily affecting the central nervous system. Several factors have been suggested to play a role in its development, including an increased exposure to sunlight (UVB), smoking, diminished levels of vitamin D, and genetic predisposition (Yousfi et al., 2023[128]; Ziemssen et al., 2019[130]). The presence of the Epstein-Barr virus (EBV) in the body of individuals has also been associated with an increased risk of MS (Filippi et al., 2020[36]; Vaughn et al., 2019[118]), though research findings on its role remain contradictory (Lassmann, 2019[73]). Recent research highlights the significance of EBV-induced B-cell transformation in MS development (Kuhlmann et al., 2023[71]). 

Clinically, MS is categorized into two forms: relapsing or progressive. The majority of the patients (approximately 85 %) express the relapsing-remitting type characterized by cycles of attacks and recovery phases in the nervous system. These attacks can vary in severity, with about 15 % of MS patients experiencing a relapse-free course, while another 15 % endure severe MS conditions (Dobson and Giovannoni, 2019[26]).

Diagnosis of MS typically relies on McDonald's criteria, which consider the presence of brain lesions and the frequency of attacks (Schwenkenbecher et al., 2019[107]). Several studies have explored the impact of the immune system on individuals with MS. Among immune cells, CD4+ T cells are recognized as key players in MS pathogenesis (Høglund and Maghazachi, 2014[57]; Kasper and Shoemaker, 2010[65]), with prolonged release of interferon gamma (IFN-γ) contributing to inflammation (Vartanian et al., 1995[117]). Among the family of T cells, T helper 17 (Th17) cells also play an important role (Volpe et al., 2015[121]), secreting pro-inflammatory cytokines IL-17 and IL-6, regulated by IL-23 (Luchtman et al., 2014[77]; Vilisaar et al., 2015[119]). It has been observed that T helper 1 ("Th1") leads to the secretion of IFN-γ (Hohnoki et al., 1998[58]; Volpe et al., 2015[121]) and Th17 cells, leading to the release of IL-17 (Bettelli et al., 2008[12]), with the increase of IL-17 in the brain leading to demyelination. 

**Figure 1:**
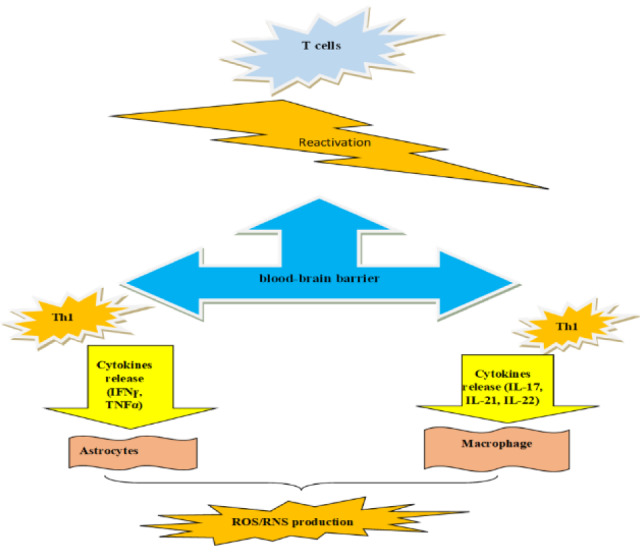
Multiple sclerosis pathogenesis in production ROS

The innate immune system can play an important role in the initiation and progression of MS by affecting T and B cells (Gandhi et al., 2010[40]; Hemmer et al., 2015[56]). These immune factors activate cytokines and other markers, leading to the activation of innate immune cells and further progression of MS (Mayo et al., 2012[81]). The following figure shows this relationship (Figure 1(Fig. 1)). 

Cytokines, a group of factors that regulate the immune response, play a crucial role in MS physiopathology (Grigoriadis and Van Pesch, 2015[46]; Navikas and Link, 1996[87]). In a healthy individual, there exists a balance between T helper and cytokines. In MS, however, this balance is disturbed, leading to an increase in pro-inflammatory Th1 cytokines (e.g. IL-1, IL-6, TNF-α) and a decrease in regulatory cytokines such as IL-10 (Amedei et al., 2012[3]). This imbalance contributes to neuronal and nervous system damage, ultimately manifesting as MS symptoms (Friese et al., 2014[39]; Ghasemi et al., 2017[41]). 

Recent research suggests that exercise training can help reduce physical disability and brain atrophy in individuals with MS by regulating cytokine production (Alvarenga-Filho et al., 2016[2]; Kjølhede et al., 2016[68]). Inflammation triggers a cascade of biochemical and cellular mechanisms (Scarisbrick, 2008[104]), triggering oxidative stress (Morel et al., 2015[85]), axonal degeneration (Correale et al., 2019[20]), and brain damage in MS patients (Kotelnikova et al., 2017[70]). The innate immune system emerges as a therapeutic target in the question to understand MS pathology (Weiner, 2008[123]). In examining the pathology of MS, researchers have observed the accumulation of numerous factors ultimately contributing to an individual's disability (Lassmann et al., 2012[74]). 

Several important physiological and functional benefits of exercise training could be listed in our manuscript about MS, including: improved aerobic capacity (Rampello et al., 2007[99]), better balance (Yang et al., 2019[126]), improved mood (with potential reduction in depression) (Grossman et al., 2010[47]), increased muscle strength (Halabchi et al., 2017[51]), enhanced quality of life (Silverman et al., 2017[110]), improvements in the immune and hormonal system (Schulz et al., 2004[106]), and fatigue reduction (Asano and Finlayson, 2014[7]). Despite these potential benefits, only a small number of individuals with MS (20 %) engage in regular physical activity (Fasczewski et al., 2018[34]), and with increasing age, the duration of exercise in MS patients tends to decrease (Pau et al., 2020[91]). Thus, we aimed to examine the existing literature to assess the benefits and potential drawbacks of exercise for MS patients, shedding light on the factors that influence exercise participation. Through this comprehensive review, we aspire to provide valuable insights that may guide future research and clinical practice in the realm of MS management and care.

## Exercise Training

Regular exercise has been proven to be a beneficial intervention for individuals with MS. First, research has consistently demonstrated that individuals with MS have lower levels of physical fitness when compared to their healthy counterparts. This discrepancy is largely due to their physical inactivity and unhealthy lifestyles (Barnard et al., 2020[10]; Döring et al., 2012[27]). Indeed, a direct relationship has been established between physical inactivity, MS, and the subsequent development of functional and physiological impairments, ultimately leading to premature fatigue and a diminished ability to participate in physical activities (Langeskov-Christensen et al., 2017[72]). Fortunately Latimer-Cheung et al. (2013[76]) have shown the effectiveness of exercise training in improving the quality of life for individuals with MS. In this context, previous studies from Grover (2016[48]) and Ponichtera-Mulcare (1993[95]) identified several factors that affect the adaptations of MS patients to exercise training, including cardiorespiratory fitness, voluntary function, skeletal muscle function, and the impact of exercise-induced temperature changes such as heat sensitivity. Therefore, it is crucial for individuals with MS to engage in regular exercise to improve their physical fitness and overall well-being (Riemann-Lorenz et al., 2020[103]). 

## Physiological and Functional Response of MS Patients to Exercise

### Neuroplasticity-induced changes in Multiple Sclerosis patients

**Figure 2:**
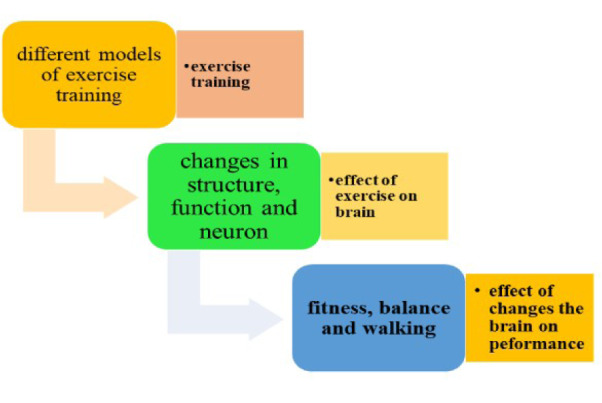
The effect of exercise training on sports performance caused by changes in the brain in MS patients

Exercise training induces beneficial effects on the central nervous system (CNS) in individuals with MS (Guo et al., 2020[50]). These changes can be observed through the reconstruction of brain structure, function and connectivity, ultimately resulting in improved physical and cognitive performances (White and Castellano, 2008[124]). For instance, long-term walking has a positive impact on both cognitive function and brain morphology of MS patients (Devasahayam et al., 2017[23]; Erickson et al., 2019[29]). The effectiveness of exercise training in inducing brain changes has been demonstrated through brain imaging (Ji et al., 2021[61]). Studies have shown that exercise induces adaptations in the brain, and these changes have been observed in MRI (Rasova et al., 2015[100]). These results can be seen in Figure 2(Fig. 2).

By affecting synapses through the activation of different brain areas, exercise can improve brain function (Prosperini et al., 2015[97]). Additionally, exercise induces the activation of the thalamus-motor cortex network, and an improvement of interneuron communication which is related to the changes that result from exercise training at the level of brain structure and at the molecular/cellular level, such as the increase of neurotrophic factors and angiogenesis in MS patients (Diechmann et al., 2021[24]). 

### The effect of exercise on the blood-brain barrier

Regular long-term exercise training improved the antioxidant capacity and decreased the levels of active oxygen species (Bogdanis et al., 2013[14]). These processes contribute to the improvement of blood-brain barrier function and reduction in its permeability to metabolites (Mokhtarzade et al., 2018[84]). By examining the effects of high-intensity interval training on the physiological indicators of the blood-brain barrier (Mokhtarzade et al., 2018[84]) (MMP-2 and MMP-9), a significant decrease in these indicators has been observed (Mokhtarzade et al., 2018[84]; Zimmer et al., 2018[131]). Physiological factors that play an important role in inflammatory processes and can affect the blood-brain bar-rier include MMP-2, for example, which could be reduced by the anti-inflammatory properties of physical training (Zimmer et al., 2018[131]). This factor can disrupt the blood-brain barrier. In a neurodegenerative disease such as MS, the permeability of the BBB is altered, and exercise can reduce the factors involved in this process, such as MMP-2, leading to an improvement in cognitive function (Zimmer et al., 2018[131]).

**Figure 3:**
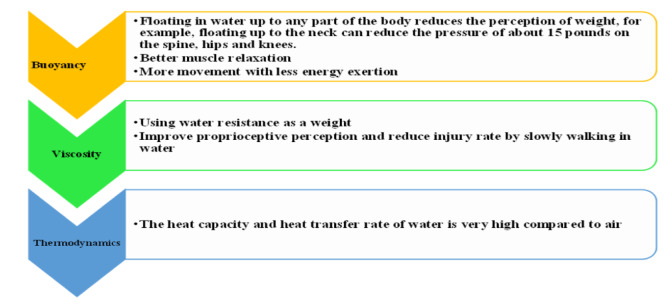
Advantages of floating in water on patients with MS

Exercise training increases the neurotrophic factors' levels such as BDNF, resulting in improved cognitive performance (Shobeiri et al., 2022[108]). Further research is needed to investigate the effects of exercise training intensity and duration on the levels of those neurotrophic factors in individuals with MS (Banitalebi et al., 2020[8]). Studies have demonstrated that aquatic exercise leads to a reduction in catecholamine levels, confirming the impact of exercise on the sympathetic and parasympathetic states (Bansi et al., 2013[9]). Figure 3(Fig. 3) shows the effect of water exercises on patients with MS.

Various factors affect the level of neurotrophic factors such as BDNF which might be affected by exercise training, inflammation, metabolic state of the body, body weight (Mokhtarzade et al., 2018[84]). In many neurological diseases such as Alzheimer's, Parkinson's, MS, and Huntington's, a lot of neurodegeneration occurs in the brain, which is accompanied by the death of neuronal nuclei and neuronal structures (Manoharan et al., 2016[79]). Many studies have shown that the combination of physical activity and rich environment such as nutritional or cognitive interventions can help in reducing, inhibiting or delaying the progress of neurodegenerative diseases (Bhatti et al., 2020[13]; Pang and Hannan, 2013[89]). Meanwhile, BDNF neurotrophic factors are very important in protecting these neurons. Natural interventions, such as exercise, hormonal balance (e.g., steroid hormones such as cortisol and testosterone), and nutritional interventions (e.g., fasting, low-calorie intake, low-carbohydrate diet, selective nutrient intake), can reduce this level and increase the neurotrophic factor and this helps the plasticity of the brain (Waterhouse and Xu, 2009[122]).

### Cytokine modulation with exercise training

Exercise training enhances immune system function by regulating and modulating cytokines (Alvarenga-Filho et al., 2016[2]; Faramarzi et al., 2020[33]). A study by Smith et al. (Smith et al., 2016[111]) examined the impact of 6 months of aerobic training on the secretion of interferon γ cytokine in patients and found a decrease in its level. Similarly, Moghadasi et al. (2015[83]) investigated the effect of 12 weeks of resistance training on the level of interleukin 6 and observed a decrease as well. Short-term exercise has been shown to maintain immune homeostasis by regulating cytokines, such as, C-reactive protein (CRP), IL-1, IL-6, IFNγ, with an increase in the level of IL-10 (Afzal et al., 2020[1]; Barry et al., 2016[11]). This cytokine regulation may have implications for the prevention and treatment of MS (Asano and Finlayson, 2014[7]). Short-term exercise training induces immune system homeostasis through cytokines' release (Fasczewski et al., 2018[34]), assisting in coping with immune system disorders during this process (Fasczewski et al., 2018[34]). While the precise mechanisms of this flow are yet to fully proven, studies suggest regular aerobic exercise induces changes in the levels of Th1 and Th2 family cytokines, potentially aiding in the treatment and control of MS (Lassmann et al., 2012[74]; Pau et al., 2020[91])

### Regulation of stress hormones

In MS, there exists a complex relationship between the endocrine glands and the immune system (Moynihan and Moore, 2010[86]). The disease involves the activation of Th1 segment leading to an excessive release of IL-12, TNFα, IFNγ and deficiency of IL-10 (Kaskow and Baecher-Allan, 2018[64]). This dysregulation can be influenced by axis stimulation and its impact on the immune system (Ysrraelit et al., 2008[129]). Notably, stress hormones can play a role in reducing demyelination in MS by altering Th2 factors (Karagkouni et al., 2013[63]). Cytokines, which are crucial in immune responses, are influenced by hormones and neural factors (Eskandari and Sternberg, 2002[31]). Catecholamines and glucocorticoids, which are two types of stress hormones, are particularly important in the production and release of cytokines (Elenkov and Chrousos, 2002[28]). Studies have shown that stress hormone regulatory systems can affect the immune system and this has been observed in patients with MS (Gold et al., 2005[43]). Regulation of these stress hormones, such as catecholamines and glucocorticoids, may reduce the extent of demyelination in MS, the mechanism being modulation of Th2 (Deckx et al., 2013[22]). As previously proven, physical activity and exercise can affect the progression of MS by modulating stress hormones (White and Castellano, 2008[125]).

### Exercise and fatigue in MS

Exercise training in patients with MS does not necessarily result in an increased perception of fatigue; rather, it can contribute to the management of fatigue. There are various reasons why exercise can lead to a reduction in the perception of fatigue in MS patient. One factor is the tendency for individuals with MS to become inactive and spend extended periods at home (Macdonald et al., 2022[78]), which can heighten the perception of fatigue whenever they will exercise afterwards, and also diminish overall performance (Kornek et al., 2000[69]). Engaging in exercise training prompts adaptation in individuals and can actually lead to an increase in delaying the perception of fatigue (Dalgas et al., 2019[21]).

## Impact of Exercise on the Innate Immune System

Exercise induces changes in body homeostasis and elicits immune and hormonal responses. Regular exercise has been shown to contribute to the amelioration of chronic inflammation of the nervous system and related factors (Florindo, 2014[37]). This beneficial outcome is achieved by shifting the status of cytokines towards their anti-inflammatory counterparts, thereby improving low-grade inflammation (Petersen and Pedersen, 2005[93]). Additionally, exercise directly impacts the innate immune system through the Toll-like receptor signal pathway. By examining the combined effects of endurance and resistance training over eight weeks in MS patients, researchers found that this combined training significantly reduced the levels of IL-17 and IFN-γ cytokines in plasma (Golzari et al., 2010[44]). IL-17 is indeed a crucial pro-inflammatory cytokine associated with the progression of MS (Stromnes et al., 2008[113]). This cytokine can break through the blood-brain barrier, which can lead to damage. The mechanism of this process is the activation of calcium release and the disruption of the integrity of the BBB (Yarlagadda et al., 2009[127]). Engaging in physical activity and participation in exercise training can exert a beneficial anti-inflammatory effect by reducing IL-17, contributing to a more favorable immune response in MS patient (Alvarenga-Filho et al., 2016[2]).

## Models of Exercise Training

When creating an exercise program for patients with MS, it is crucial to take into account the patient's status and primary training goals, which may encompass improving muscle strength, endurance, balance, coordination, and reducing exercise fatigue (Ertekin et al., 2012[30]; Halabchi et al., 2017[51]). The design of the exercise program should carefully consider all elements, including frequency, intensity, duration, methods employed, and obviously, necessary precautions (Platta et al., 2016[94]). Various exercise programs have been recommended for individuals with MS, encompassing strength training, endurance training, balance training, and aquatic exercise programs. Each program can be customized to address the specific needs of the individual patient and should be supervised by a qualified healthcare professional to ensure safety and effectiveness (Döring et al., 2012[27]; Kalb et al., 2020[62]). Below, we will outline some exercise programs that could be prescribed for these patients.

A protocol commonly employed for individuals with MS involves exercising in stairs (Taylor et al., 2006[115]). This model is effective since the level of the test execution is equal to the ground level, and it is especially suitable for MS patients (Brown and Kraft, 2005[17]). It is recommended that individuals with MS perform these exercises at least once daily. The execution involves climbing stairs within the range of motion that they do not feel pain, and this type of exercise is typically well-suited for patients with mild disabilities. Stair exercises encompass a blend of strength, endurance, flexibility, balance and coordination exercises (Sieljacks et al., 2020[109]). These exercises contribute to reduction of perceived fatigue and improvement in the quality of life (Carpinella et al., 2018[18]). When incorporating other exercises alongside the staircase model, it is crucial to assess the individual needs and abilities of the patient. Additionally, complementing stair training with exercises in water/pool can serve as a beneficial supplement (Brody and Geigle, 2009[16]).

### Aerobic training

Engaging in aerobic exercises by individuals with MS has been shown to lead to improvements in psychological factors, quality of life and aerobic capacity, furthermore, it has been to be generally well-tolerated and safe (Swank et al., 2013[114]). Studies suggest that a four-week aerobic exercise protocol can yield favorable gains in cardiorespiratory fitness for MS patients (Andreu-Caravaca et al., 2021[4]). Recommended exercises include performing light activities on a cadence bike and/or a treadmill with sufficient support (Latimer-Cheung et al., 2013[76]). Many studies utilizing a treadmill have reported significant positive outcomes in MS patients (Latimer-Cheung et al., 2013[76]). The recommended aerobic training program is 2 to 5 sessions per week (Döring et al., 2012[27]; Snyder, 2017[112]). Exercises intensity is suggested to be around 40-70 % of VO_2max_, with the percentage of maximum heart rate at approximately 60-80 (or 40-60 % of heart rate reserve) (Asadi, 2022[5]; Humphreys, 2022[59]). Depending on the progression of the disease, the duration of training is planned in intervals of 10 to 40 minutes, divided into three segments of ten minutes each (Latimer-Cheung et al., 2013[76]). The rate of progression and training intensity serves as a key factor in the initial six months of training planning (Halabchi et al., 2017[51]).

Engaging in resistance exercises for individuals with MS necessitates the involvement of an experienced trainer to ensure both safety and effective performance monitoring (Barry et al., 2016[11]; Vartanian et al., 1995[117]). The resistance training program for MS patients should incorporate equipment and weight machines that minimize risks (Vartanian et al., 1995[117]). In cases where traditional bodybuilding machines are not available/feasible, using elastic bands or body weight exercises as resistance can be a suitable alternative (Bettelli et al., 2008[12]; Vartanian et al., 1995[117]). A training frequency of 2 to 3 gym sessions per week is considered appropriate for MS patients. Each session should be designed with 8 to 15 repetitions maximum (RM) at an intensity ranging from 60 % to 80 % of 1RM (Devasahayam et al., 2017[23]; Erickson et al., 2019[29]; Rasova et al., 2015[100]). Gradual increases in training intensity is strongly recommended (Bettelli et al., 2008[12]; Lassmann, 2019[73]), with a suggested load incremental of 2 to 5 % in each session to promote progress while preventing injuries (Bettelli et al., 2008[12]; Vartanian et al., 1995[117]).

Concerning the flexibility and stretching exercises, typically individuals with MS experience a decrease in joint range of motion due to inactivity. Flexibility exercises are a dependable and low-impact solution, contributing to increased muscle length, reduced muscle spasms, enhanced joint mobility, and improvements in balance and body posture (Bettelli et al., 2008[12]). In a training program, these exercises are recommended daily for 10 to 15 minutes (Bettelli et al., 2008[12]; Karagkouni et al., 2013[63]; Ysrraelit et al., 2008[129]). It is advisable to incorporate these movements both before and most importantly after the training session, encompassing both upper and lower body movements (Devasahayam et al., 2017[23]; Erickson et al., 2019[29]; Rasova et al., 2015[100]).

In individuals with MS, where the central nervous system is impacted, a heightened focus on enhancing balance and coordination is essential. These exercises involve the manipulation of their center of gravity. An effective method for improving coordination and balance is the Swiss ball exercise, which contributes to increased strength and flexibility (Halabchi et al., 2017[51]). Additionally, incorporating tai chi exercises with deliberately slow movements is another beneficial approach to enhance strength and balance. Water-based exercises are also noteworthy for increasing balance and flexibility, while reducing the risk of falling (Eyvaz et al., 2018[32]).

Respiratory muscle training is aimed at enhancing the efficiency of the respiratory muscles during exercise, paralleling the improvement seen in skeletal muscles (Gosselink et al., 2000[45]). In their examination of fatigue factors in individuals with MS, Foglio et al. (1994[38]) noted that respiratory muscle weakness could be one of these contributing factors to the rapid fatigue of patients with MS. In addition, improvement in these muscles has been observed following respiratory muscle training in people with MS, supporting the potential for using this technique as complementary training (Martín-Valero et al., 2014[80]).

### Physiological response to exercise in MS

The cardiovascular system's response to exercise activities is carried out by the autonomic nervous system. Research investigating the impact of exercise training on the cardiovascular response in individuals with MS has identified slight abnormalities at rest (Feltham et al., 2013[35]; Keytsman et al., 2019[66]). Ponichtera-Mulcare (1993[95]) conducted a study comparing the cardiovascular response to exercise in MS patients and those with physical disability. It is interesting to note that they did not find a significant difference in the cardiovascular response to exercise, because the severity of the disease is a key determinant in the response of the cardiovascular system to exercise in patients with MS (Heine et al., 2016[55]).

Aerobic capacity of MS patients has been demonstrated to decline, with researchers primarily attributing it to reduced engagement in physical activities (Proschinger et al., 2022[96]). Additionally, respiratory dysfunction may play a role in diminishing performance (Comi et al., 2001[19]), as compromised respiratory muscles can result in decreased respiratory function (Induruwa et al., 2012[60]). Weakened in muscles affected by central nervous system (CNS) dysfunction becomes apparent during maximal exercise tests, and this weakening and atrophy can stem from short-term non-use or spinal cord injury in individuals with MS (Dionyssiotis et al., 2014[25]). Studies have identified a lower presence of type I muscle fibers, along with a reduction in muscle size and enzyme activity, among MS patients who are not actively engaged in physical activities (Hansen et al., 2015[52]; Petajan and White, 1999[92]). This decline contributes to a decrease in functional capacity, highlighting the significance of implementing an exercise program to enhance muscles strength (Hansen et al., 2015[52]). 

### Signaling pathway involved in the impact of physical activity in MS

Various mechanisms contribute to the effects of physical activity on individuals with MS, including the reduction of fat tissue leading to diminished adipokines and the augmentation of an anti-inflammatory environment (Negaresh et al., 2018[88]). It is noteworthy that exercise training, irrespective of weight loss, serves as an intervention in chronic inflammatory diseases by influencing cytokines and adipokines, as highlighted in studies by Gleeson et al. (2011[42]) and Negaresh et al. (2018[88]).

In addition to the effect of exercise on physiological and molecular issues in patients with MS, improving the quality of life is a crucial aspect for individuals with MS, given the limited availability of curative treatments. Quality of life serves as a measure of an individual's happiness and overall enjoyment life (Barry et al., 2016[11]; Langeskov-Christensen et al., 2017[72]). Due to the lack of treatment solutions in patients with MS, improving the condition and quality of life of these patients can play an effective role in reducing stress and dampening the disease' symptoms (Schroeder et al., 2014[105]). Various studies indicate that individuals with MS experience adversity on multiple fronts (Borkoles et al., 2008[15]; Pust et al., 2020[98]). Research exploring the quality of life among MS patients reveals a notable trend that these individuals frequently experience with a lower quality of life compared to those with other conditions, even in comparison to patients with intestinal inflammation (Guerrero Aznar et al., 2022[49]; Visser et al., 2021[120]). This decline in life satisfaction is particularly pronounced in the early years of the onset of the disease (Patten et al., 2012[90]).

**Figure 4:**
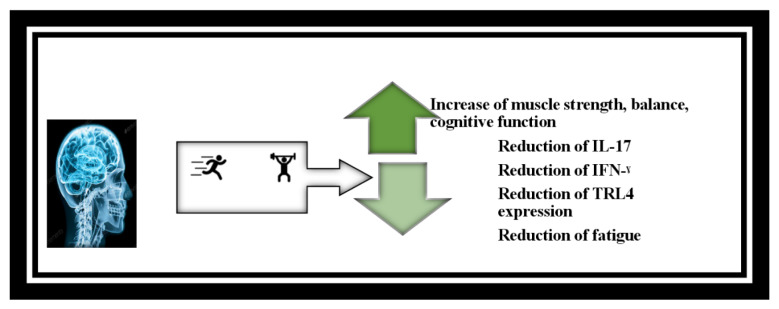
Effect of exercise training on MS patient

Encouraging and persuading individuals affected by loneliness and depression to engage in regular activities for fitness and interpersonal connections is crucial. Exercise training practices play a significant role in reducing feeling of loneliness and depression (Tollár et al., 2020[116]). The type and diversity of exercises has been shown to impact the quality of life for patients with MS (Heesen et al., 2006[54]; Tollár et al., 2020[116]). Among various exercise modalities, aerobic exercises have proven to be more effective, leading to an enhancement in these patients' overall quality of life (Andreu-Caravaca et al., 2021[4]). Studies investigating the duration of exercise have shown that two to three days a week are recommended, starting with two days a week and ending with three days a week. Each training session should be ten to thirty minutes, starting from ten minutes and progressing to thirty minutes. The intensity of each session can be between ten and thirteen on a twenty-point scale, or between 40 and 60 percent of the maximum oxygen consumption (VO_2_peak) or maximum heart rate (HR_max_). Overall improvement should begin with increasing duration or frequency. The method of training progress should be based on tolerance (Kalb et al., 2020[62]; Kim et al., 2019[67]). Figure 4(Fig. 4) illustrates the impact exercise on MS.

## Prescription of Exercise Training for MS

Researchers have designed guidelines and protocols for engaging individuals with MS in physical activities based on essential investigations dedicated to those patients. These studies consistently advocate the incorporation of moderate-intensity aerobic exercises, spanning at least, 30 per session, and two days a week. Additionally, to enhance muscle strength, participation in strength exercises for two other days a week is recommended (Asano et al., 2009[6]; Latimer-Cheung et al., 2013[75]). Alternative protocols for strength training in individuals with MS involve focusing on major muscle groups, typically comprising 1-3 sets with 8 to 15 repetitions at maximum capacity (Asano et al., 2009[6]). Exercise recommendations for MS patients at different stages are shown in Table 1(Tab. 1) (Kalb et al., 2020[62]).

We also believe that diet can substantially improve life by instigating changes and modifications, given its crucial role in metabolism and its status as a fundamental life resource. The substances within our diet, influencing energy production, play a crucial role in the catabolic and anabolic processes of our body (Riccio and Rossano, 2015[101]; Riccio et al., 2010[102]). It is worth nothing that the components present in these dietary compounds can wield significant influence over the onset or prevention of various diseases. However, it is imperative to underscore that a comprehensive understanding of the topic will necessitate future research (Diechmann et al., 2021[24]).

## Conclusion

According to the information presented in this article, participation in sports training leads to improved quality of life, reduced perception of fatigue, improved hormonal levels and immune system function in people with MS. Doing training exercises creates motivation, vitality, happiness and life expectancy in patients with MS, because these people refuse to do physical activities as soon as they find out about the disease. Exercising can have a positive effect on mechanisms related to brain function through neurotrophic factors such as BDNF in maintaining brain integrity. The promising link between physical activity, brain health, and improved hormonal and immune status in MS patients highlights the need for further research.

## Notes

Saber Saedmocheshi and Narimen Yousfi contributed equally as first author.

## Declaration

### Contributions conceptualization

SS, KC, NY: Methodology; writing -original draft preparation; review and editing.. All authors have read and agreed to the published version of the manuscript. 

### Data availability statement 

Not applicable.

### Declaration of conflicting interests 

The author(s) declare no potential conflicts of interest with respect to the research, authorship, and/or publication of this article.

### Funding

The author(s) received no financial support for the research, authorship, and/or publication of this article. 

### Informed consent statement 

Not applicable. 

### Institutional review board statement 

Not applicable. 

## Figures and Tables

**Table 1:**
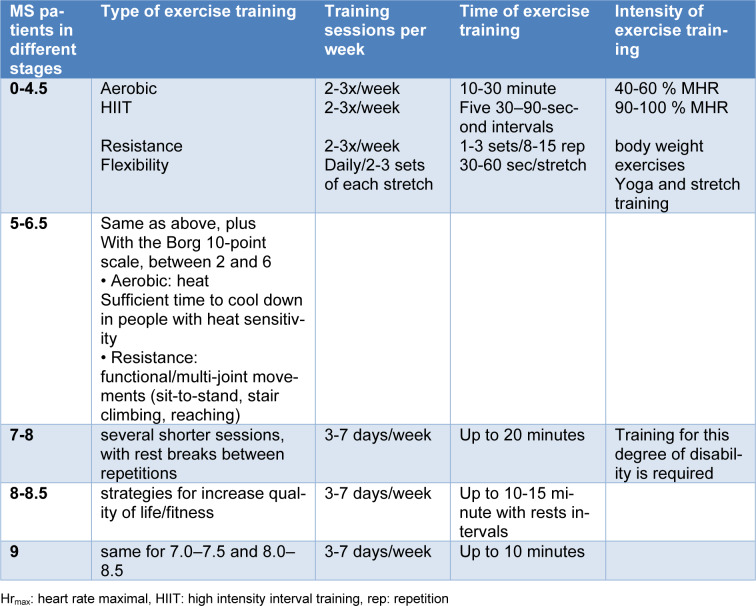
Exercise recommendations for Expanded Disability Status Scale 0-9.0
